# Supplementary material for: Genome editing for scalable production of alloantigen‐free lentiviral vectors for in vivo gene therapy
Source: EMBO Mol Med. 2017 Aug 23;9(11):1558–73. doi: 10.15252/emmm.201708148 (PMC5666310; doi:10.15252/emmm.201708148)
Supplement: Supplementary file 1 — Appendix [file EMMM-9-1558-s001.pdf]

Genome Editing for Scalable Production of Alloantigen-free Lentiviral Vectors for *in Vivo* Gene Therapy

Alloantigen-free Lentiviral Vectors

Michela Milani[1,2], Andrea Annoni[1], Sara Bartolaccini[1], Mauro Biffi[1], Fabio Russo[1], Tiziano Di Tomaso[1], Andrea Raimondi[3], Johannes Lengler[4], Michael C. Holmes[5], Friedrich Scheiflinger[4], Angelo Lombardo[1,2], Alessio Cantore[1*], Luigi Naldini[1,2*]

[1]San Raffaele Telethon Institute for Gene Therapy, IRCCS San Raffaele Scientific Institute, Milan 20132, Italy;

[2]Vita Salute San Raffaele University, Milan 20132, Italy;

[3]IRCCS San Raffaele Scientific Institute, Milan 20132, Italy;

[4]Baxalta (former Baxter) Innovation GmbH, Vienna 1221, Austria;

[5]Sangamo Therapeutics, Inc, Richmond CA 94804, USA;

[*]These authors share senior authorship

Corresponding author: Luigi Naldini, San Raffaele Telethon Institute for Gene Therapy, Via Olgettina 58, 20132, Milan, Italy; luigi.naldini@hsr.it

Table of Contents

Appendix Figure S1

Appendix Figure S1

A

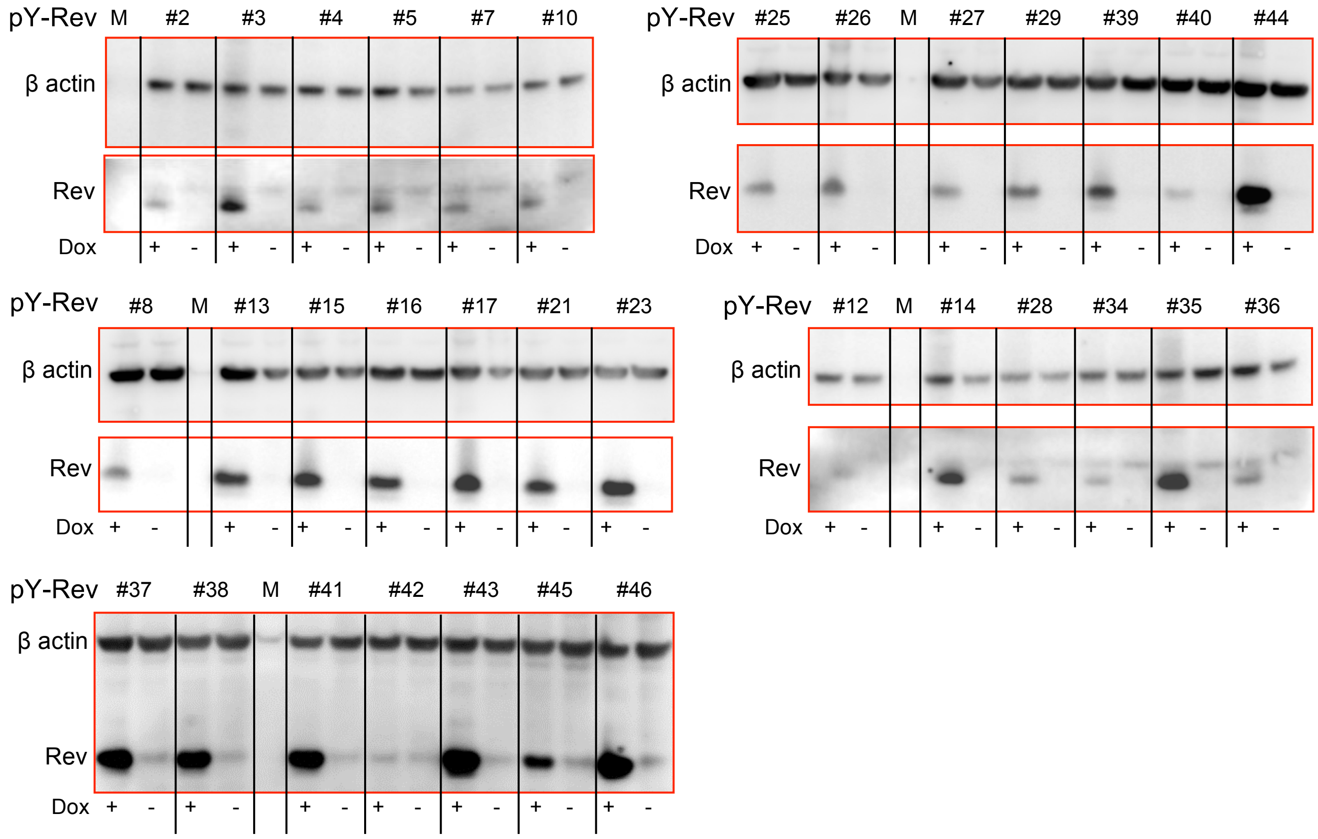

B

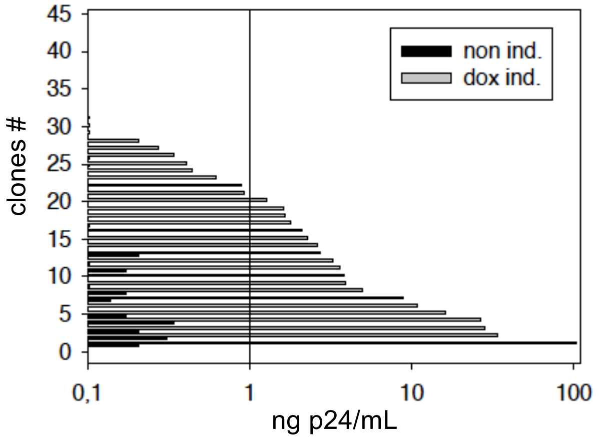

C

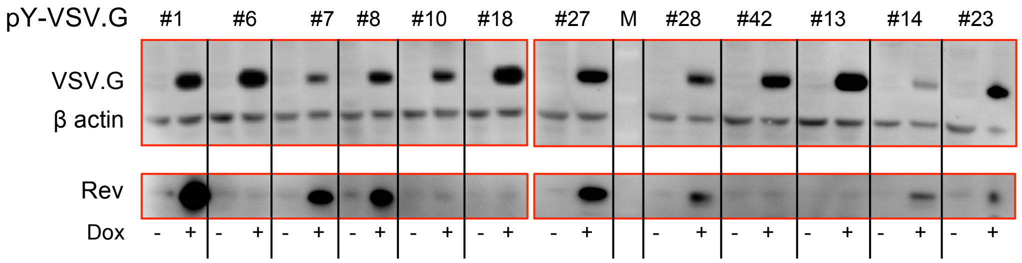

Appendix Figure S1 - Generation of LV packaging cell line.

A Western blot on protein extracts from single-cell clones obtained from 293 T-REx stably transfected with pY-Rev, induced (+) or not (-) with dox. Red borders show images taken from different blots.

B LV particles production (ng p24/mL) in the medium of single-cell clones obtained from 293 T-Rex Rev stably transfected with pY-Gag/Pol induced (grey bars) or not (black bars) with dox.

C Western blot on protein extracts from single-cell clones obtained from semi-packaging cell line stably transfected with pY-VSV.G, induced (+) or not (-) with dox. Red borders show images taken from different blots or gels.
