# Supplementary material for: Genome editing for scalable production of alloantigen‐free lentiviral vectors for in vivo gene therapy
Source: EMBO Mol Med. 2017 Aug 23;9(11):1558–73. doi: 10.15252/emmm.201708148 (PMC5666310; doi:10.15252/emmm.201708148)
Supplement: Supplementary file 2 — Expanded View Figures PDF [file EMMM-9-1558-s002.pdf]

Expanded View Figures

Figure EV1. Generation of LV producer cell lines.

A Schematic representation of the plasmid used as donor DNA (pLV) for homologous recombination to target the LV genome transfer constructs used in this work into the *AAVS1* (see also Fig 1E). Brown and light blue arrows represent the sequences homologous to the genomic target site, respectively. PGK, phosphoglycerate kinase promoter; ET, enhanced transthyretin promoter (Cantore *et al*, 2015); FIX, coagulation factor IX, co-FIX-Padua, codon-optimized hyper-functional FIX transgene (Cantore *et al*, 2015); 142T, four tandem repeats of microRNA 142 target sequences (Cantore *et al*, 2015). The brown and light blue arrows represent the primers used to detect the LV genome junctions (see Fig 1F).

B Percentage of GFP-positive cells (mean with range or single value, n = 1–2), in LV packaging cell line transiently transfected with the indicated amount of ZFN-expressing plasmid and 1 μg of the indicated donor plasmid and analyzed by flow cytometry, 2 weeks after transfection.

C Percentage of GFP-positive cells (black bars, left y-axis) and MFI (gray bars, right y-axis) in bulk GFP-positive (+) or GFP-negative (−) sorted populations and single-cell clones obtained from three independent T.I. experiments performed with the indicated donor DNA, analyzed by flow cytometry at least 1 month after sorting.

D, E DNA copies of *Rev* (pink bars), *Gag* (gray bars), or *VSV.G* (blue bars) per diploid genome (D) and ZFN copies (DNA copies of *FOK*) per diploid genome (E) in untreated 293T cells (UNT), bulk GFP-positive (+) or GFP-negative (−) sorted populations and single-cell clones obtained from three independent T.I. experiments performed with the indicated donor DNA.

© 2017 The Authors

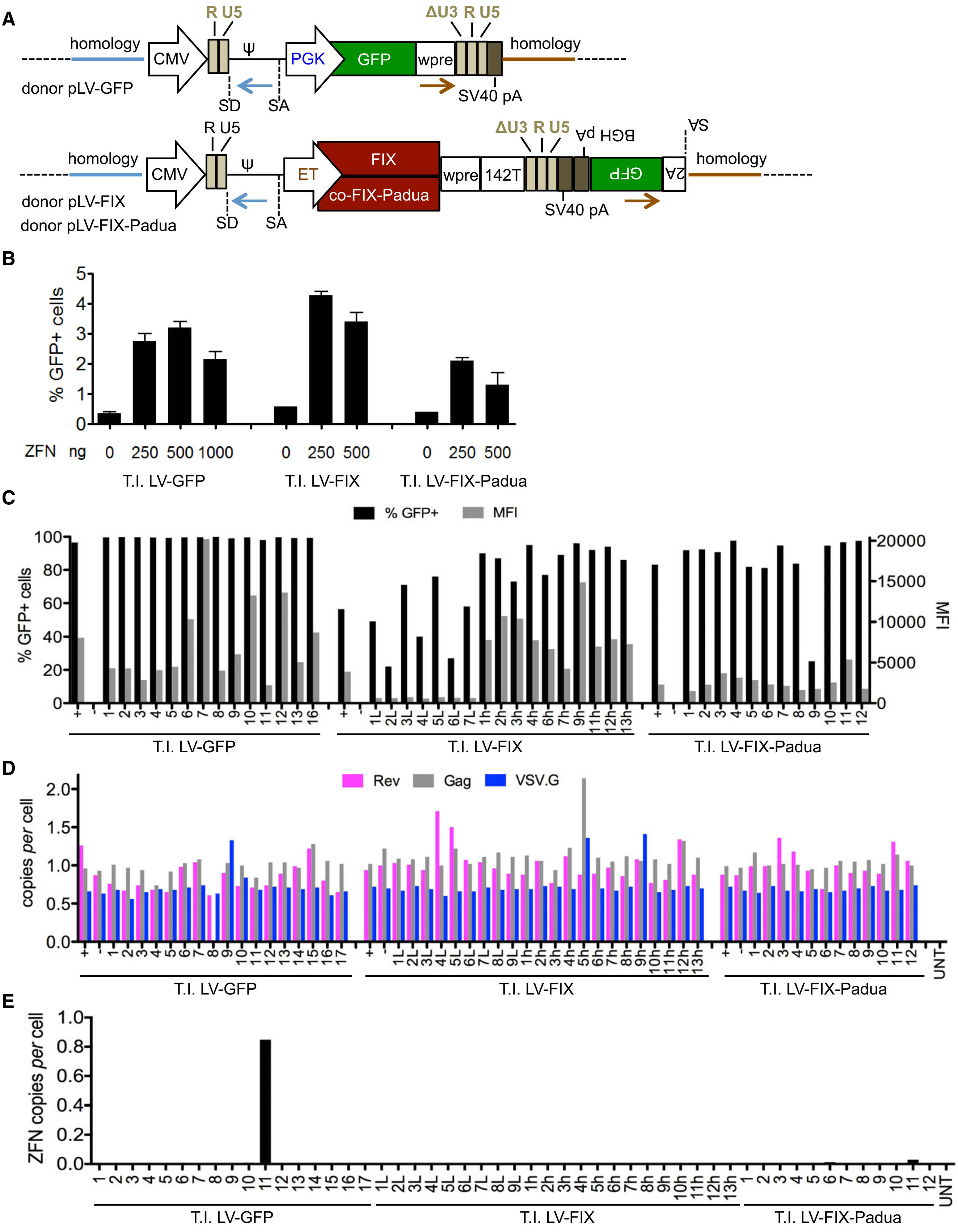

Figure EV1.

 © 2017 The Authors

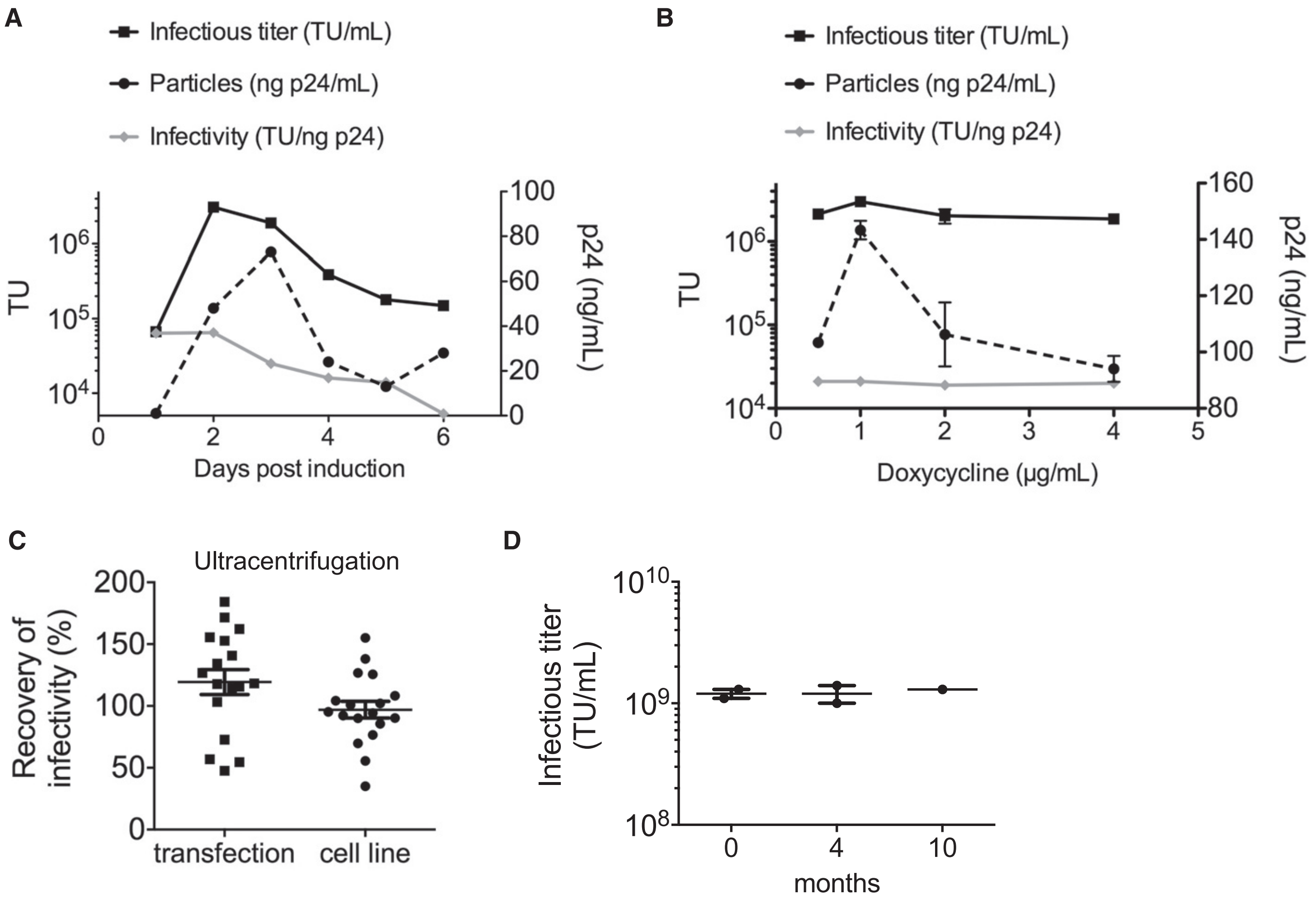

Figure EV2. Yield and stability of cell line-produced LV.

A, B LV infectious titer (TU/ml, black line, plotted on left *y*-axis), physical particles (ng p24/ml, dashed line, plotted on right *y*-axis) and specific infectivity (TU/ng p24, gray line, plotted on left *y*-axis) in (A) conditioned medium of LV-GFP producer cell line, collected at the indicated day after dox induction (n = 1 per time point), and in (B) conditioned medium of LV-GFP producer cell line induced at the indicated dox concentration, collected 3 days after induction (n = 3 per dox concentration, mean with SEM).

C Percentage of recovery of infectivity (single values and mean with SEM) upon ultracentrifugation of LV produced by cell lines (circles, n = 18) or of LV produced by transient transfection (squares, n = 17).

D Infectious titer (TU/ml; single values and mean or mean with range, n = 1–2) of two concentrated LV produced by LV-GFP producer cell line, determined at the indicated time (months) upon storage at −80°C.

Figure EV3. HSPC and T-cell transduction.

A Gating strategy for HSPC and CFC population analysis. FMO: fluorescence minus one; arrows indicate sub-gating from the previously gated population.

B, C Mean with SEM (MOI 10 and 100, n = 4) or range (MOI 300, n = 2) of the CFC composition in (B) erythroid progenitors (CD235a-positive cells) and (C) myeloid progenitors (CD33-positive cells), 2 weeks after transduction with LV produced by transient transfection (white bars) or by LV-GFP producer cell line (black bars), expressed as fold change to untreated cells (UNT).

D Gating strategy for T-cell population analysis.

E, F Mean with SEM ("cell line", n = 4) or range ("transfection", n = 2) of the population composition in CD4-positive (E) or CD8-positive (F) cells, 1 week after LV transduction, expressed as fold change to untreated cells (UNT).

▶

© 2017 The Authors

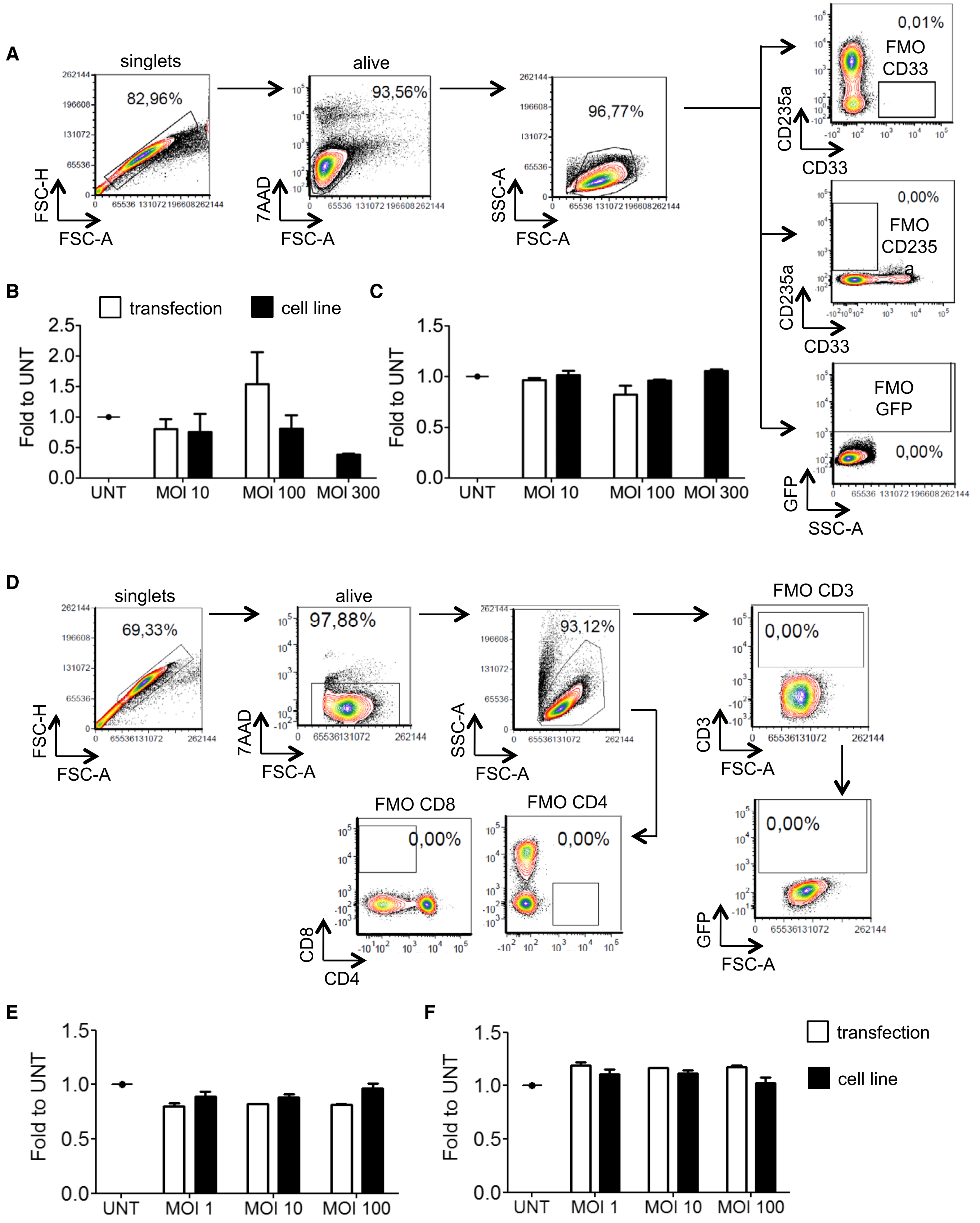

Figure EV3.

© 2017 The Authors

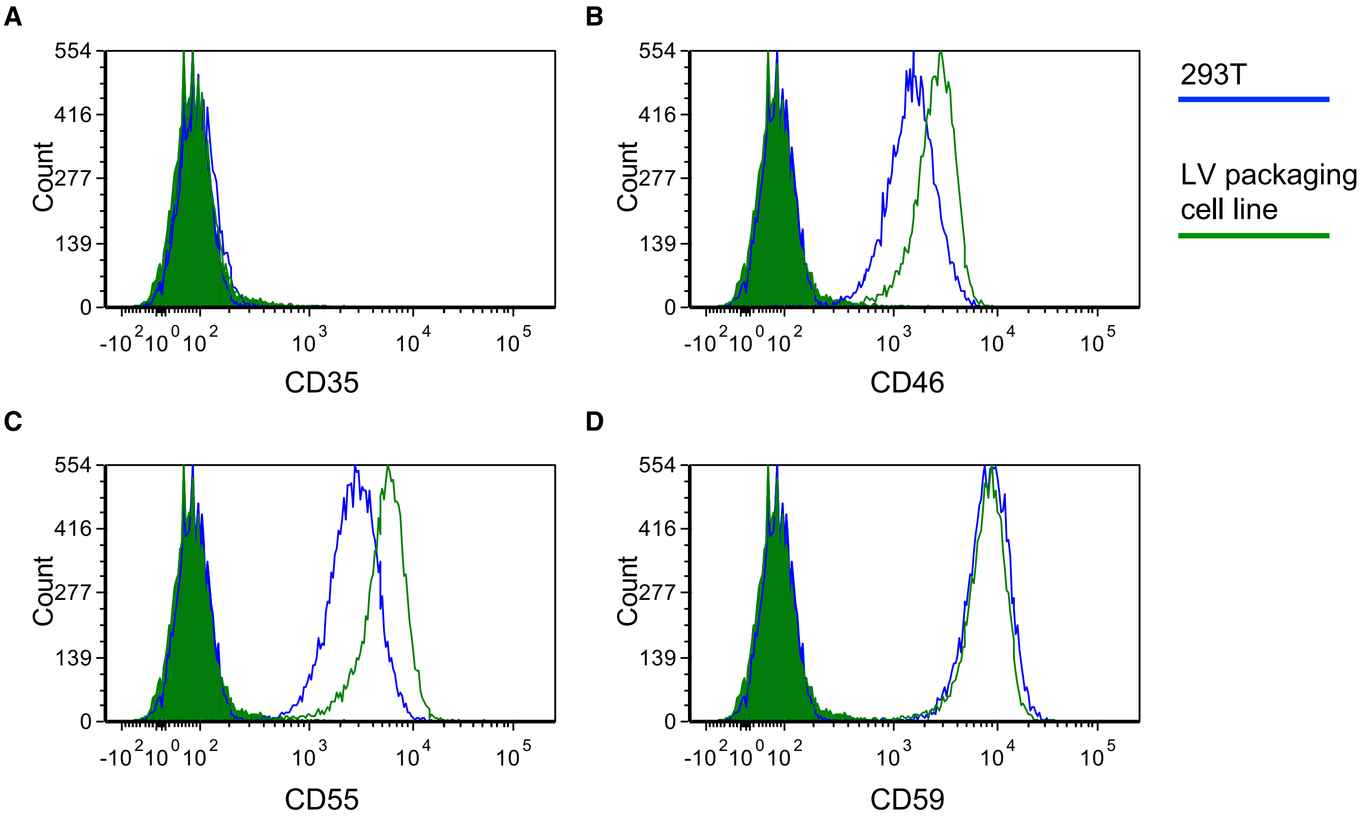

Figure EV4. Expression of complement regulatory proteins on LV producer cells.

A–D Flow cytometry analysis (histograms) of 293T (blue line) or LV packaging cell line (green line), unstained (filled lines) or stained (empty lines) with anti-CD35 (A), anti-CD46 (B), anti-CD55 (C), anti-CD59 (D) antibodies, as indicated.

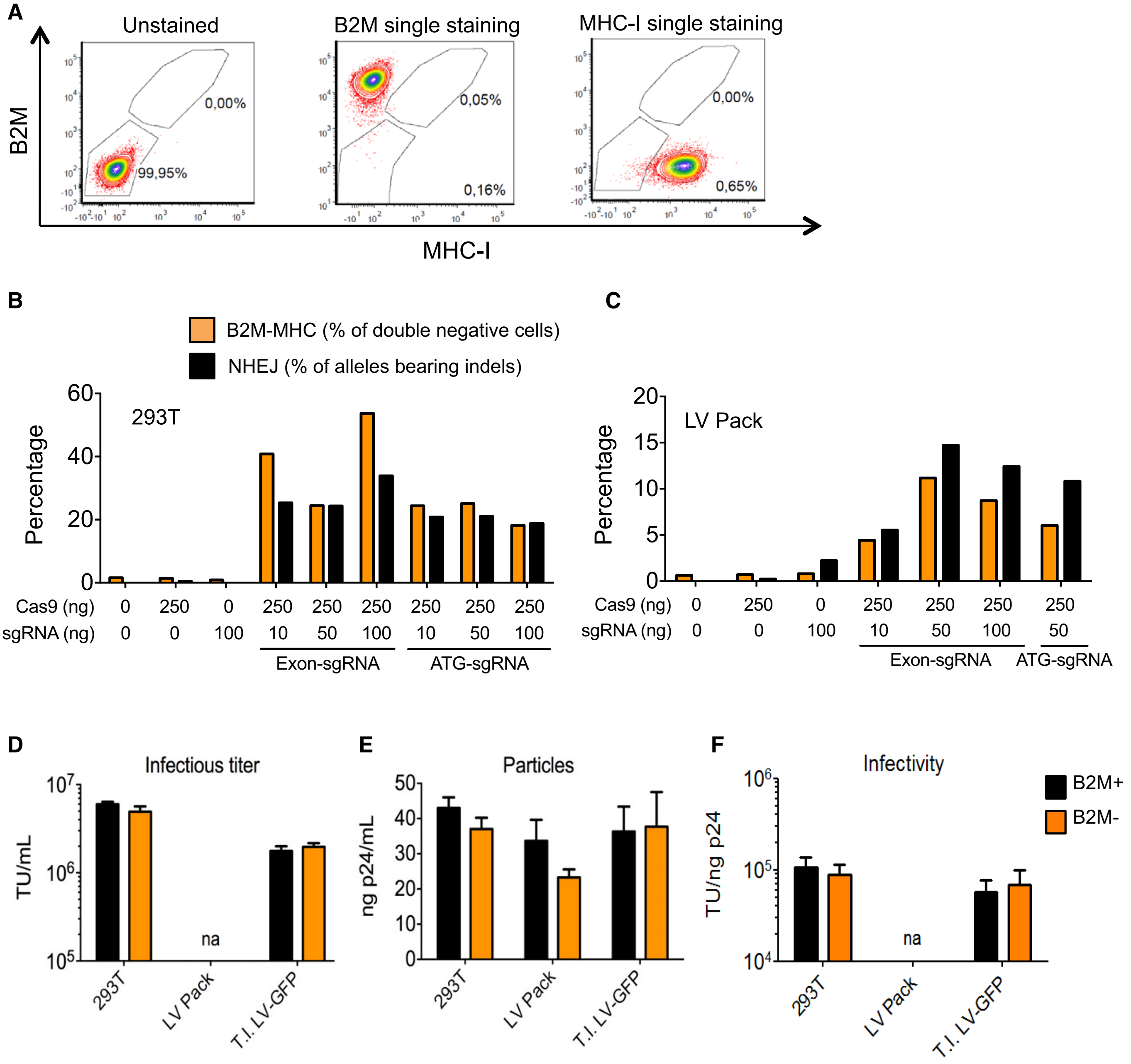

Figure EV5. Generation of B2M-negative cells.

A Gating strategy for MHC-I negative and B2M-negative cells.

B, C Percentage of B2M- and MHC-I-negative cells (orange bars) and of alleles bearing indels (NHEJ, black bars) in 293T (B) or LV packaging cell line (C) transiently transfected with the two different sgRNAs (exon 1 or ATG directed as indicated) with the indicated quantities of Cas9 and sgRNA-expressing plasmids, 1 month after transfection.

D–F Mean with SEM of (D) infectious titer (TU/ml), (E) physical particles (ng p24/ml), and (F) specific infectivity (TU/ng p24) of LV produced by B2M-positive (black bars, *n* = 3) or by B2M-negative (orange bars, *n* = 3) 293T, LV packaging cell line or LV-GFP producer cell line as indicated. No significant differences by Mann–Whitney test. “na”, not available.

 © 2017 The Authors
