# Supplementary figures and images for: Genome editing for scalable production of alloantigen‐free lentiviral vectors for in vivo gene therapy
Source: EMBO Mol Med. 2017 Aug 23;9(11):1558–73. doi: 10.15252/emmm.201708148 (PMC5666310; doi:10.15252/emmm.201708148)

Source Data, DNA Electrophoresis Gels, Fig 1F

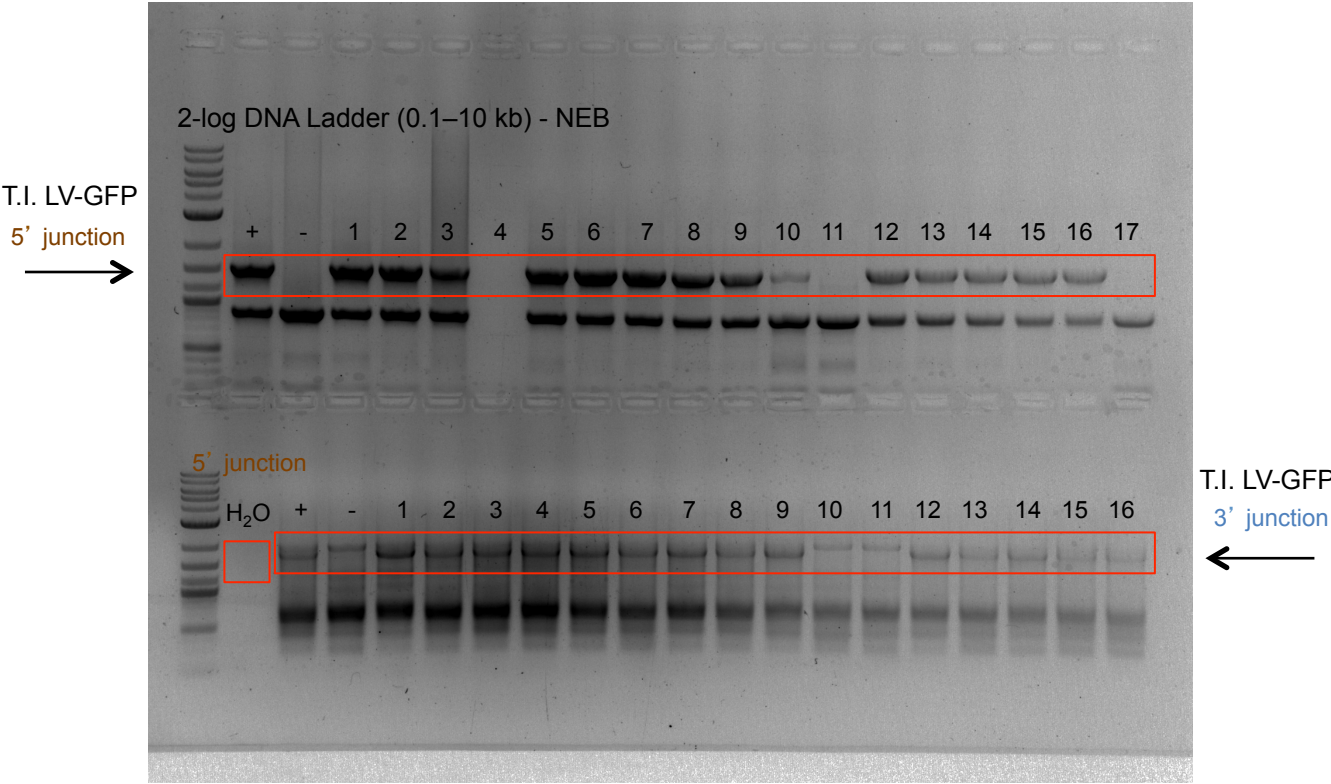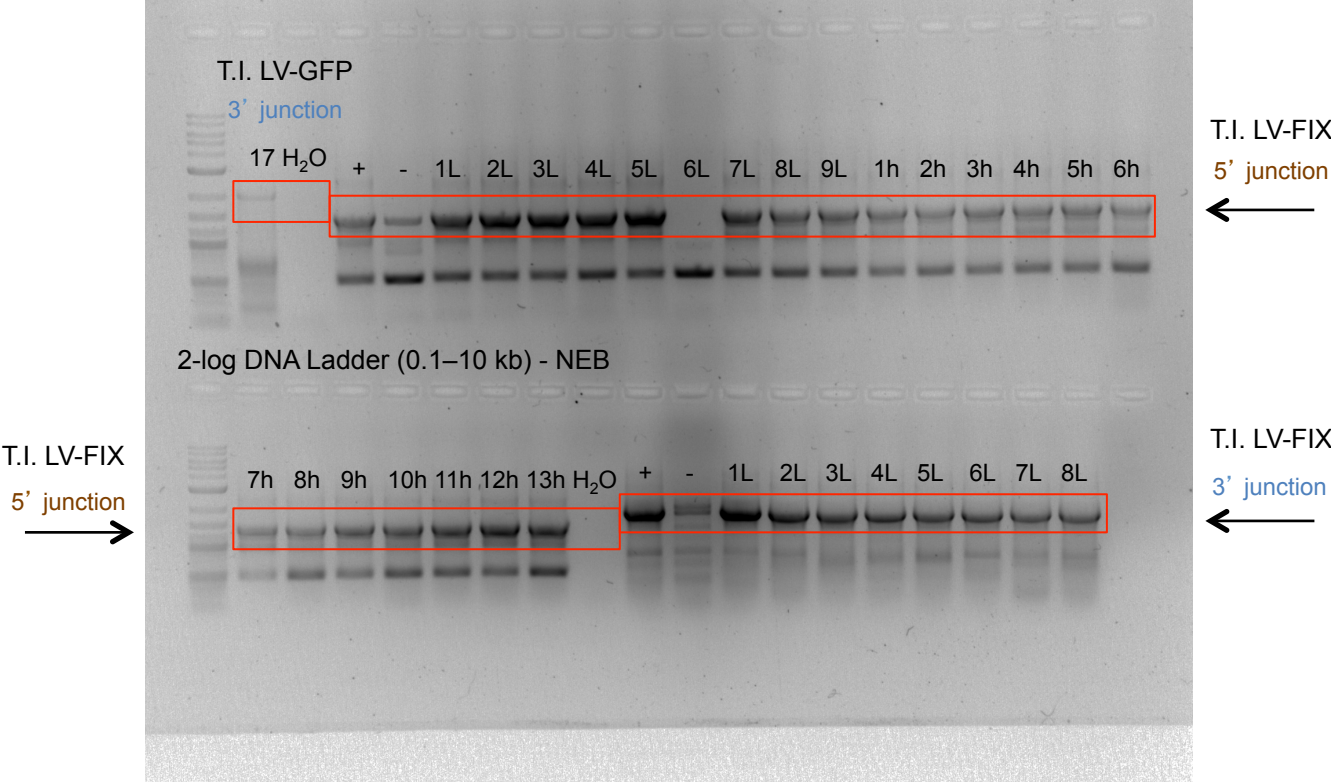

### Source Data, DNA Electrophoresis Gels, Fig 1F

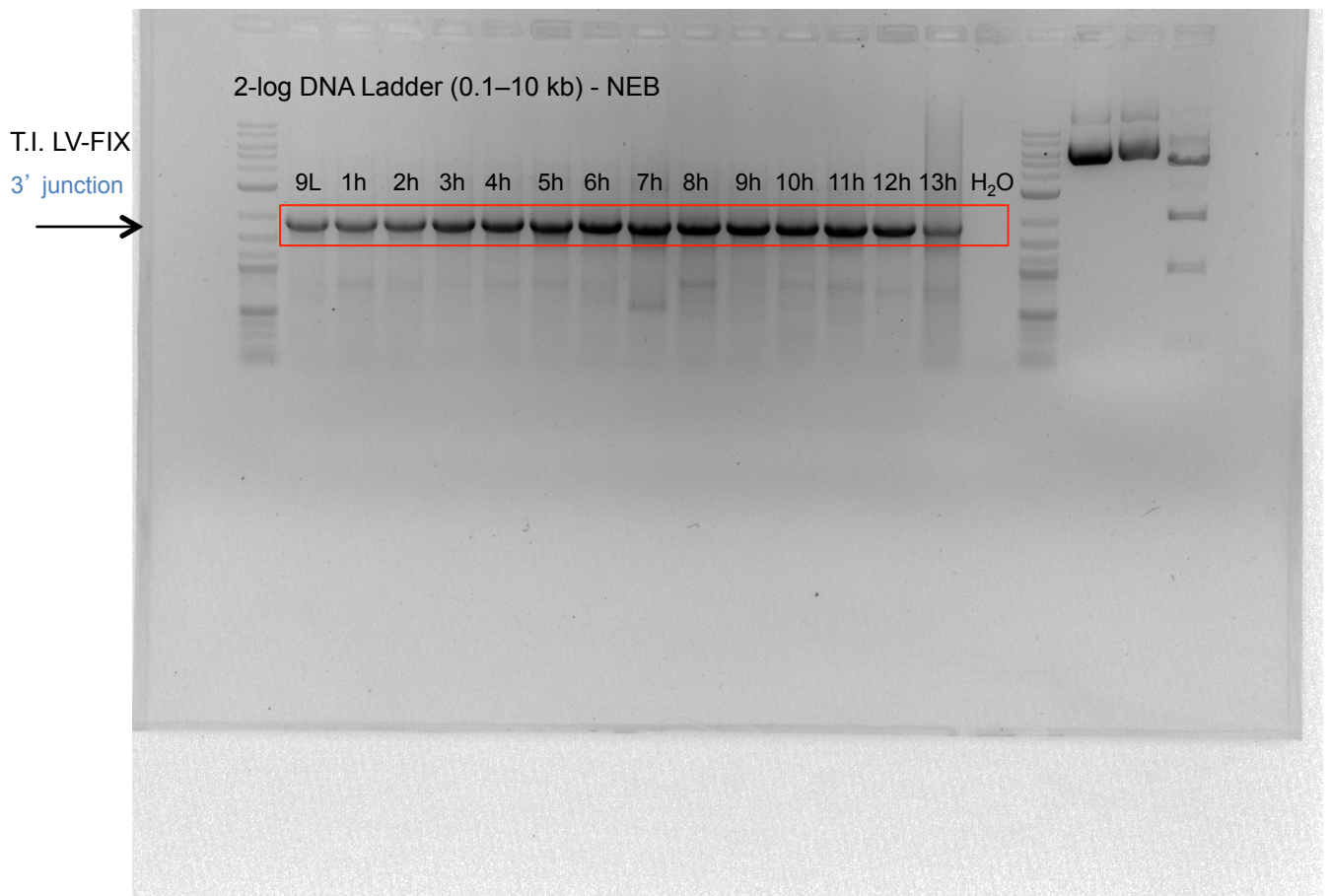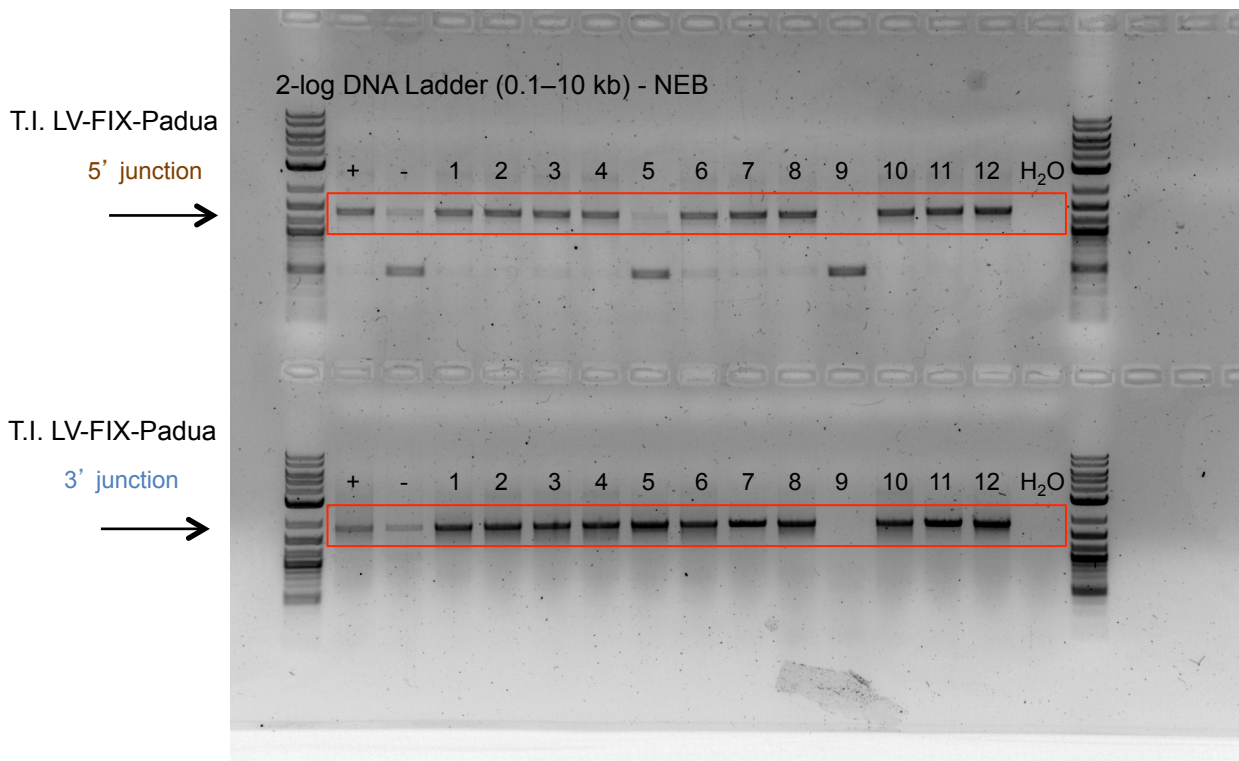

Supplement: Supplementary file 4 — Source Data for Figure 1 [file EMMM-9-1558-s003.pdf]

# Source Data, Western Blots, Fig 4A

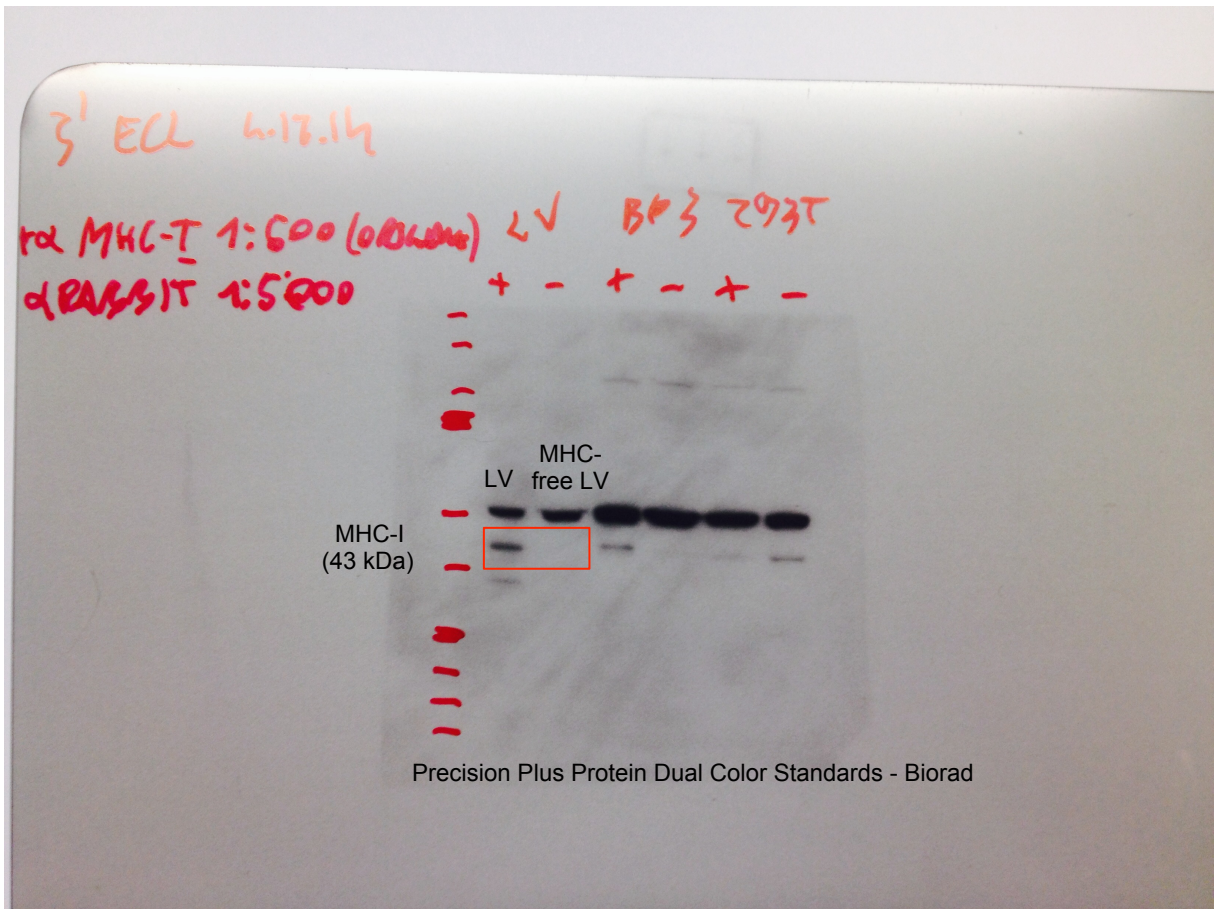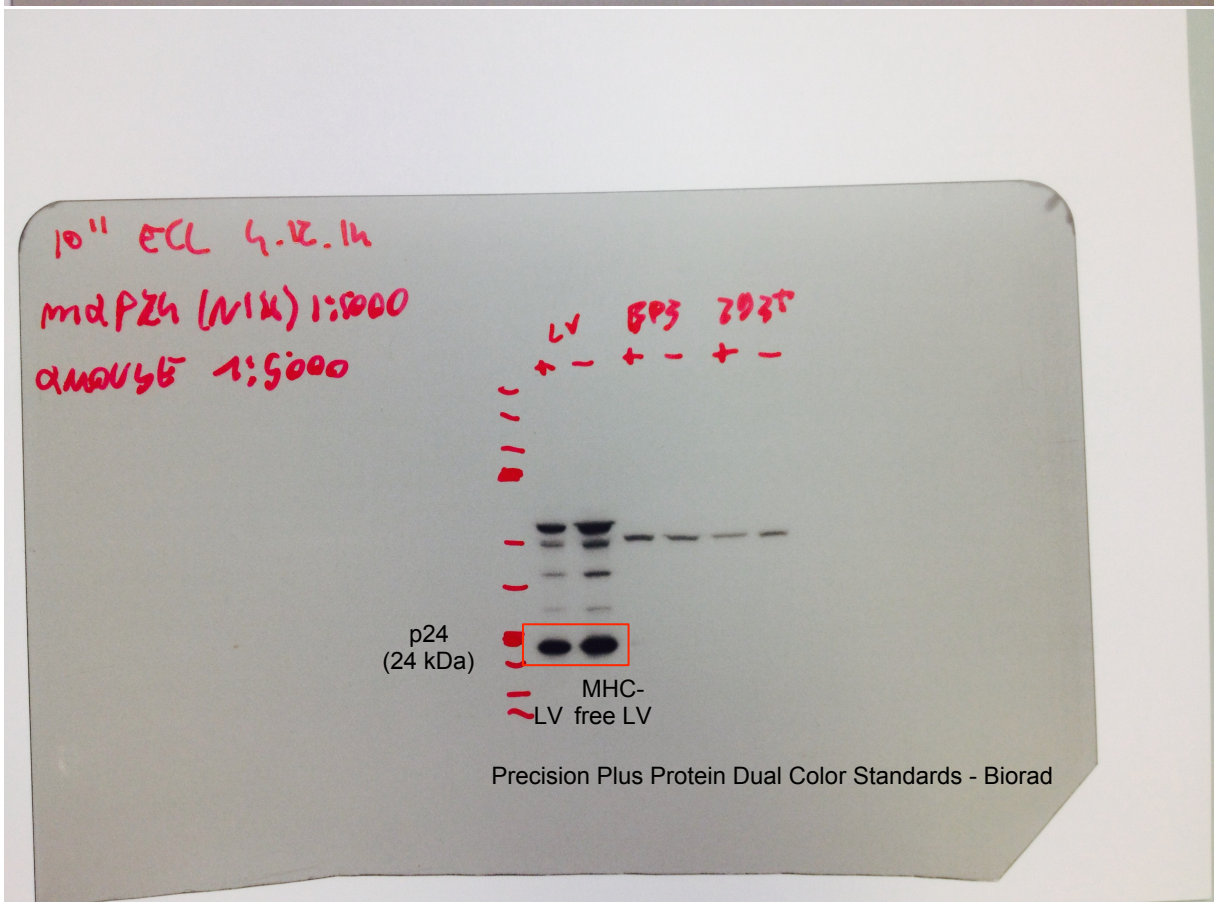

Supplement: Supplementary file 5 — Source Data for Figure 4 [file EMMM-9-1558-s004.pdf]
